# MicroRNA miR-204 and miR-1236 inhibit hepatitis B virus replication via two different mechanisms

**DOI:** 10.1038/srep34740

**Published:** 2016-10-13

**Authors:** Jyun-Yuan Huang, Hung-Lin Chen, Chiaho Shih

**Affiliations:** 1 Institute of Biomedical Sciences, Academia Sinica, Taipei, Taiwan

## Abstract

Hepatitis B virus (HBV) is a major human pathogen. In this study, we found that miR-204 and miR-1236 were down-regulated in HBV-producing cells, and each could suppress HBV replication. Using a bioinformatic approach and a reporter assay, we identified miR-1236, which can reduce HBV replication and protein production by directly targeting at HBV specific mRNA. In contrast, miR-204 was identified by a microarray approach, and had no effect on HBV RNA and protein production. Surprisingly, miR-204 could inhibit HBV pregenomic RNA encapsidation and capsid assembly. We further demonstrated that HBV suppressed miR-204 expression via activating a host transcription factor STAT3. We established a positive feed-forward loop between HBV, miR-204 and STAT3. Interestingly, miR-204 has been considered as a tumor suppressor in some literature. Since the risk for hepatocellular carcinoma (HCC) is significantly increased in chronic HBV patients, it is possible that chronic suppression of miR-204 by HBV contributes to HCC incidence. Both miR-204 and miR-1236 might be useful for developing new therapeutics against HBV.

Hepatitis B virus (HBV) is an enveloped hepatotropic DNA virus belonging to the *Hepadnaviridae* family. Chronic infection with HBV leads to liver fibrosis, cirrhosis, and malignant hepatocellular carcinoma (HCC). To date, there are more than 240 million chronic HBV carriers worldwide. Once HBV infects human hepatocytes, its relaxed circular (RC) DNA genome can translocate into the nucleus, and the RC DNA can then convert into covalently closed circular DNA (cccDNA). HBV cccDNA template can use cellular transcriptional machinery to produce viral mRNA and proteins. While HBV vaccine is effective and treatment for HBV is available, it has remained a challenge to completely eradicate the virus from patients^1^.

MicroRNA (miRNA) is a short RNA molecule with 22–24 nt in length. They are one kind of non-coding RNAs, which are unable to encode proteins. MiRNAs control gene expression by degrading or suppressing the translation of target mRNAs^2^. The targeting specificity is mediated by base-pairing between the seed sequences of the miRNAs and the complementary target sequences on the 3′-untranslated regions (3′UTR) of the targeted mRNA transcripts. MiRNAs can participate in many critical biological processes, including development, differentiation, apoptosis and proliferation. To date, there are more than 2500 identified miRNAs in *Homo sapiens* (miRBase Version 21). The involvement of microRNAs in virus-associated diseases, including HCC, has been well studied recently^3^,^4^,^5^. However, miRNAs associated with HBV replication have remained to be further investigated.

Several cellular miRNAs have been reported to influence the replication of HBV, such as miR-130a^5^, miR-125a-5p^6^, and miR-199a-5p^7^. Our previous study demonstrated that miR-130a can inhibit HBV replication and gene expression via targeting two host factors, PPARγ and PGC1α ^5^. These two factors can individually stimulate HBV enhancer/promoter transcription in hepatoma cells. In this study, we identified two cellular microRNAs, miR-1236 and miR-204 (new nomenclature miR-204-5p), both of which can suppress HBV replication, yet through totally different mechanisms. The former can directly inhibit HBV protein translation and the latter can attenuate HBV capsid assembly and RNA encapsidation. These two microRNAs are potential candidates for antiviral therapy. Finally, these two successful approaches to microRNA hunting could be generally applicable to other non-HBV systems.

## Results

**Figure 1:**
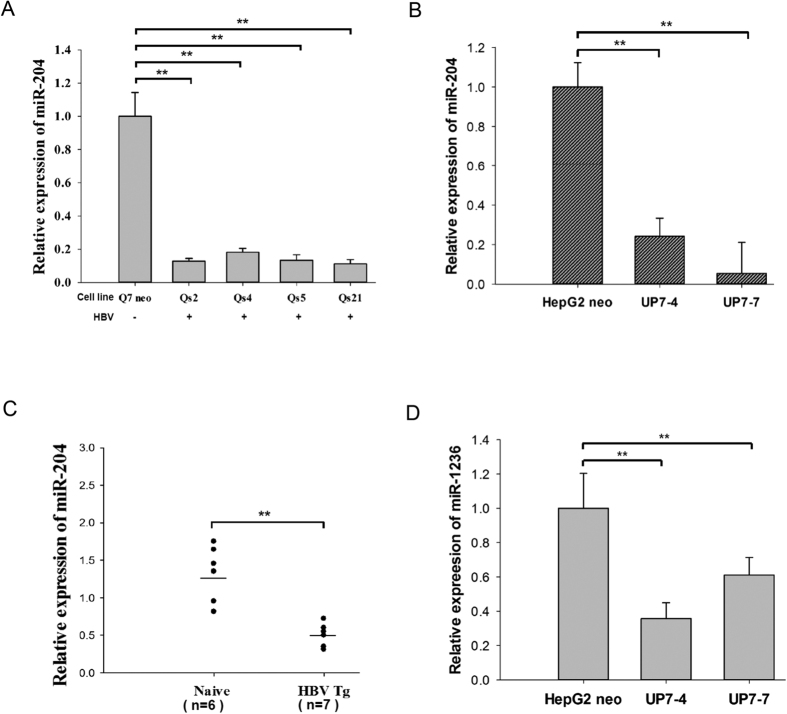
Expression of miR-204 and miR-1236 were reduced in HBV-replicating hepatocytes and HBV-transgenic mice. The expression level of miR-204 was always significantly reduced in stable (**A**) rat, (**B**) human HBV-producing cell lines, and (**C**) HBV transgenic mice. (**D**) Reduced miR-1236 was only detected in human HBV-producing cell lines. **p < 0.05.

Previously, we established a series of HBV-producing rat hepatoma cell lines capable of producing infectious virions in chimpanzees^8^,^9^. To investigate the potential relationship between HBV and cellular miRNAs, we compared microRNA expression profiles between these stable HBV-producing and control cell lines by qPCR microarray, and observed significant reduction of at least a dozen microRNAs in HBV-producing cells^8^. By stem-loop qPCR analysis, we validated that the expression of miR-204 was indeed decreased in HBV-producing rat and human hepatocytes (Fig. 1A,B). Furthermore, miR-204 was also significantly reduced in an HBV transgenic mouse model (genotype D, serotype *ayw*) (Fig. 1C)^10^. In a separate approach via bioinformatics analysis, we identified miR-1236 as a potential anti-HBV miRNA by using the Microinspector target-prediction algorithm. This microRNA of human origin has no counterpart in rodents. It can bind to the 3′ UTR on HBV genome with the highest free energy (−30.8 Kcal/mol) (Tables 1 and 2). Interestingly, we found that hsa-miR-1236 was also reduced in expression in human HBV-producing cell lines (Fig. 1D).

**Figure 2:**
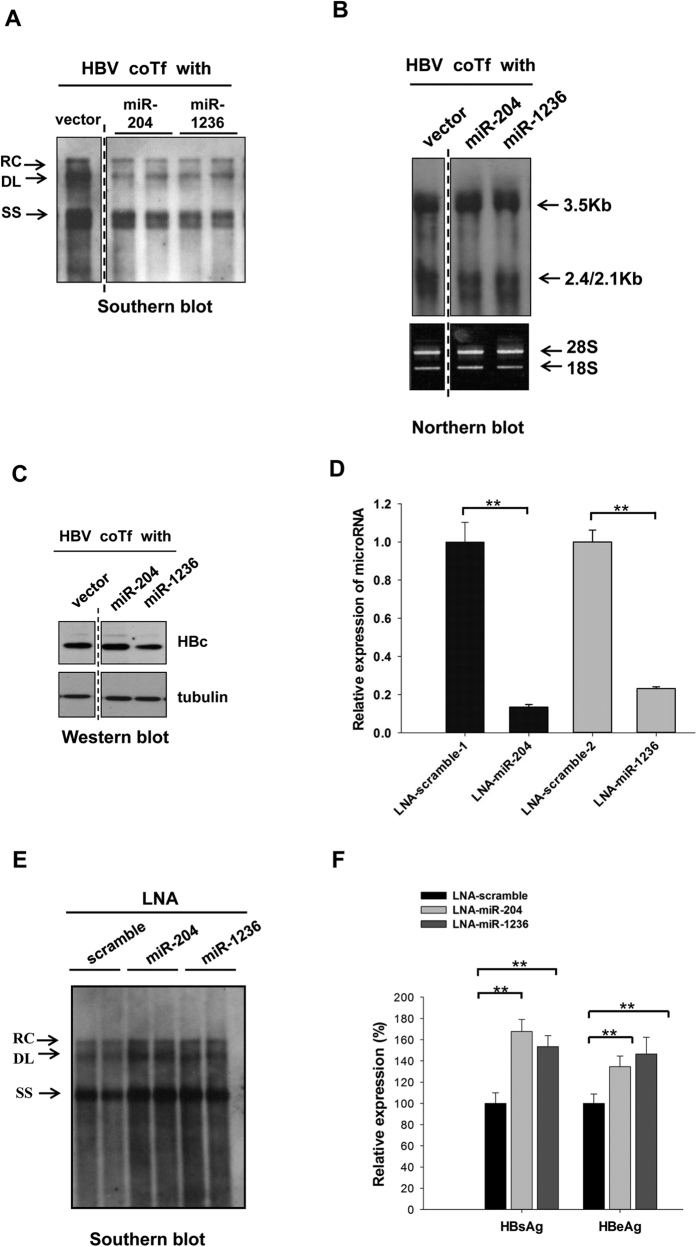
MicroRNA miR-204 and miR-1236 can each attenuate HBV replication and gene expression. (**A**) Intracellular HBV replication in HepG2 cells were reduced by cotransfection (coTf) of an HBV *ayw* genomic dimer plasmid and miR-204 or miR-1236 expression vectors using Southern blot assay. Empty vector DNA was included as a negative control. HBV replicative intermediates: RC relaxed circle, DL double-strand linear, SS single-strand viral DNA. The vertical dotted line indicates that all the lanes are spliced from the same gel. (**B**) Cotransfection with miR-204 or miR-1236 had no apparent effect on HBV precore and pregenomic RNA (3.5 Kb) and envelope-specific mRNA (2.4/2.1 Kb) by Northern blot analysis. The vertical dotted line indicates that all the lanes are spliced from the same gel. (**C**) Reduction of HBV core protein (HBc) was detected by cotransfection with miR-1236, but not by miR-204, via Western blot analysis. The vertical dotted line indicates that all the lanes are spliced from the same gel. (**D**) The reduction of endogenous miR-204 and miR-1236 by LNA treatment was measured by stem-loop qPCR. (**E**) HBV DNA replication was enhanced by LNA-miR-204 or LNA-miR-1236 treatment which can antagonize the endogenous miR-204 and miR-1236. (**F**) Secreted HBsAg and HBeAg from HBV were stimulated by LNA-antagomir cotransfection using an ELISA assay. (**p < 0.05).

We hypothesized that some of these miRNAs with reduced expression in HBV-replicating hepatocytes, could have a negative effect on HBV replication. This hypothesis could explain their reduced expression levels in several independently established rat and human HBV-producing cell lines (Fig. 1A,B). To evaluate the effect of microRNA on viral replication, we cloned the precursors of miR-204 and miR-1236 in miRNA expression vectors, respectively. HepG2 cells were cotransfected with microRNA expression vectors and an HBV *ayw* genomic dimer plasmid. Five days post-transfection, the intracellular HBV core particle-associated DNA was isolated and analyzed by Southern blot^11^,^12^,^13^,^14^. As shown in Fig. 2A, miR-204 and miR-1236 could each inhibit HBV DNA replication, without altering the level of HBV RNA expression in the cytoplasm (Fig. 2B). The expressions of miR-204 and miR-1236 were detected by stem-loop PCR analysis (Fig. S1). Next, we examined the effect of these two microRNAs on HBV protein expression, including core protein (HBc) (Fig. 2C), surface antigen (HBsAg) and e antigen (HBeAg) (data not shown). Only miR-1236 and miR-130a^5^ (data not shown), but not miR-204 and vector control, could reduce HBV protein expression. To further elucidate the mechanism, we cotransfected an HBV genomic replicon with LNA-miR-204-5p or LNA-miR-1236 into HepG2 cells (Fig. 2D). Knockdown of endogenous miR-204-5p or miR-1236 significantly resulted in increased HBV DNA replication (Fig. 2E) and protein synthesis (Fig. 2F). This LNA experiment confirmed the anti-HBV potential from miR-204-5p and miR-1236.

**Figure 3:**
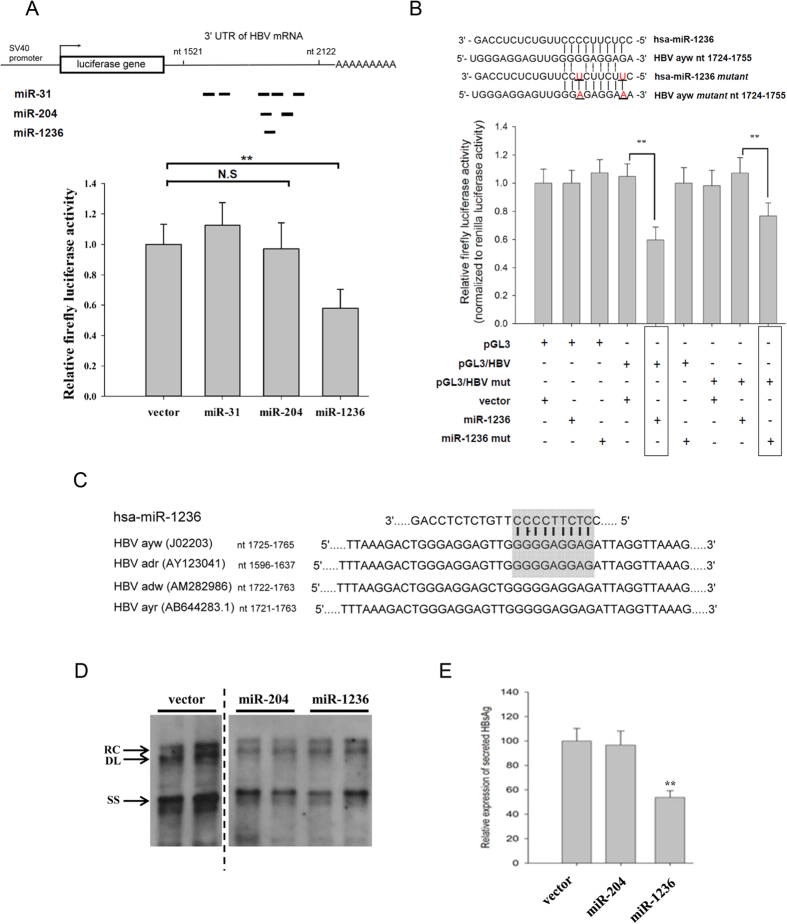
MiR-1236 can directly target at HBV specific RNA of different genotypes. (**A**) *Upper panel*: Potential microRNA target sites on HBV *ayw* genome were predicted by different computer algorithms. *Lower panel*: HuH-7 cells were cotransfected with a luciferase reporter plasmid containing HBV nt 1521-2122 and various miRNA expression vectors. Only miR-1236 displayed significant reduction of luciferase activity (**p < 0.05). (**B**) By compensatory mutagenesis, miR-1236 was shown to target HBV genome directly. *Upper panel*: Sequence alignment of wild type and mutant miR-1236 with putative target sites on wild type or mutant HBV genomes (nt 1724-1755). Mutation sites were underlined. *Lower panel*: HuH-7 cells were cotransfected with wild type or mutant pGL3-HBV 3′ UTR (nt1521-2122) reporter and wild type or mutant miR-1236 expression vector. Reduction in firefly luciferase activity was restored when mutant miR-1236 was matched with the mutant reporter (**p < 0.05). (**C**) Multiple sequence alignment of predicted hsa-miR-1236 target site on the viral genome of different HBV subtypes. Shaded box highlights the seed sequence of hsa-miR-1236 and its target site sequences. (**D**) Co-transfected miR-204 and miR-1236 significantly reduced HBV (*adr*) DNA synthesis by Southern blot analysis. (**E**) Significant reduction of HBsAg in the medium was detected by ELISA assay in the cotransfection with miR-1236, but not with miR-204.

Using Computer-aided programs, we predicted potential target sites of miR-1236 and miR-204 clustering between nt 1521 and nt 2122 on the HBV *ayw* genome (Fig. 3A). Results from the 3′ UTR luciferase reporter assay suggested that miR-1236, but not miR-204, significantly reduced the luciferase activity (Fig. 3A). We also performed compensatory mutagenesis by introducing mutations into the seed sequences of miR-1236 and its predicted target site on HBV (Fig. 3B). Only the combination of seed mutant miR-1236 and the target site mutant of HBV significantly restored the inhibitory effect of miR-1236 on the reporter activity (Fig. 3B). This result suggests a direct interaction between miR-1236 and HBV specific RNA. We also investigated whether miR-1236 can target HBV RNA of a different genotype, such as *adr*. As shown in Fig. 3C, the target sites of miR-1236 appear to be evolutionarily conserved. When the genomic dimer of HBV *adr* was cotransfected with a miR-1236 expression vector, both viral DNA synthesis (Fig. 3D) and secreted HBsAg (Fig. 3E) were significantly inhibited. Therefore, miR-1236 has a negative effect against HBV subtypes other than *ayw*.

**Figure 4:**
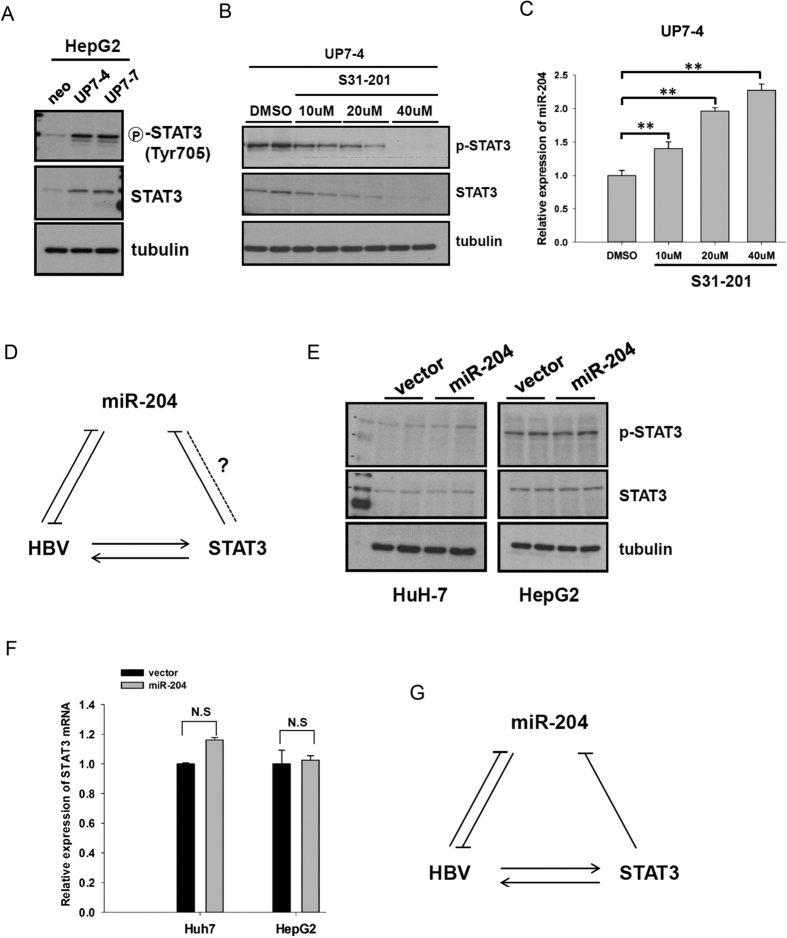
HBV repressed miR-204 expression through STAT3 activation. (**A**) Increased expression of phosphorylated and total protein levels of STAT-3 was detected in stable HBV-producing cells by Western blot with specific antibodies, (**B**) Treatment with increasing concentrations of a STAT3 inhibitor (S31-201) led to gradual reduction of phosphorylated and total STAT3 protein in stable HBV-producing HepG2 cells. (**C**) Increasing concentrations of a STAT3 inhibitor (S31-201) led to gradually increased expression of miR-204 as measured by stem-loop Q-PCR. (**D**) This cartoon summarizes the relationships among HBV, miR-204 and STAT3. The dot line and the question mark represent an unknown relationship between miR-204 and STAT3. Similar expression levels of total and phosphorylated STAT3 protein (**E**) and mRNA (**F**) were detected in HepG2 and HuH7 cell lines stably expressing miR-204. (**G**) A cartoon summarizes a positive feed-forward loop between HBV, miR-204 and STAT3. HBV can reduce the expression of miR-204 through the activation of a STAT3 intermediate.

In Fig. 1A–C, we observed that the expression of miR-204 was lower in HBV producing cell lines and transgenic mice. It is known that the expression of miR-204 can be suppressed by STAT3 activation^15^. In addition, it has been shown that HBx can activate the STAT3 signaling pathway^16^,^17^. We therefore investigated whether STAT3 can regulate miR-204 in HBV-producing hepatocytes. As shown in Fig. 4A, the expression of total and phosphorylated STAT3 proteins was strongly increased in HBV-producing cells. When we treated these HBV-producing cells with a STAT3 inhibitor, S31-201, we observed decreased levels of the total and phosphorylated STAT3 proteins in a dose dependent manner (Fig. 4B). It is therefore interesting to ask whether one can restore miR-204 expression in HBV-producing hepatocytes by treatment with S31-201. Indeed, the expression of miR-204 was gradually increased upon STAT3 inhibitor treatment (Fig. 4C). Taken together, our results demonstrated that HBV probably suppressed the expression of miR-204 through STAT3 activation. Conversely, miR-204 can inhibit HBV replication through some unknown mechanism (Fig. 4D). Is it possible miR-204 can target STAT3 in a reciprocal manner? To further explore the relationship between miR-204 and STAT3, we established two stable miR-204-expressing HuH-7 and HepG2 cells lines. As shown in Fig. 4E,F, higher expression of miR-204 driven by the CMV promoter had no apparent effect on the expression of STAT3 protein and mRNA. A positive feed-forward loop summarizes the relationships among HBV, STAT3, and miR-204 (Fig. 4G). In this triad diagram, HBV can stimulate STAT3, which in turn can suppress the level of miR-204, leading to enhanced viral gene expression replication.

**Figure 5:**
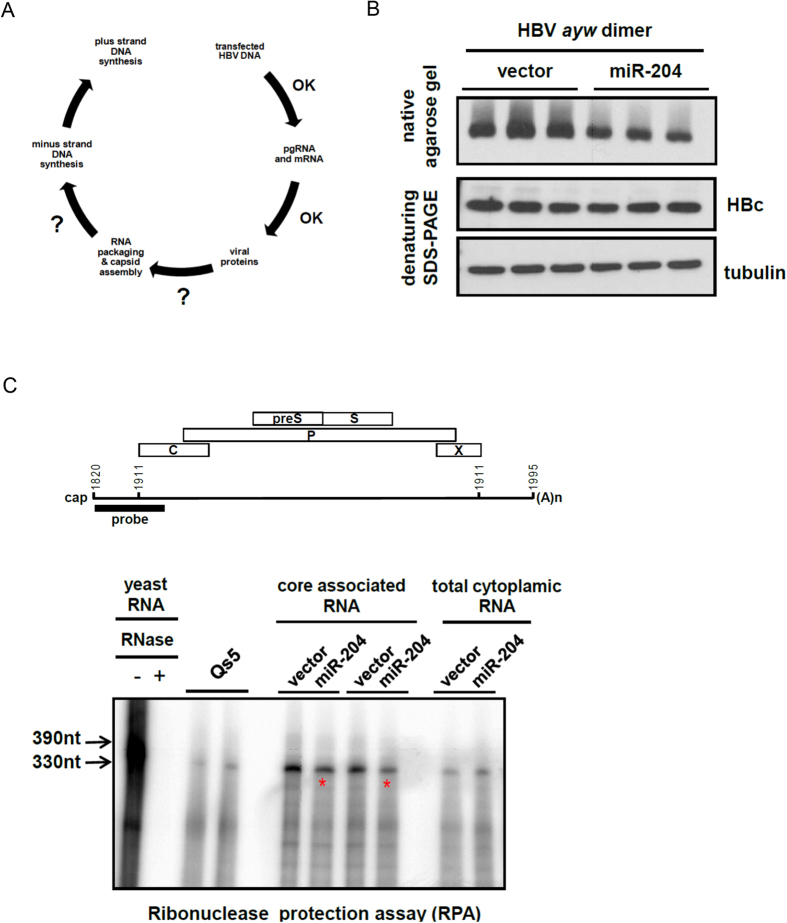
MiR-204 interfered with HBV RNA encapsidation and capsid assembly. (**A**) This diagram indicated that miR-204 could interfere with either HBV capsid assembly or reverse transcription. (**B**) *upper panel*: HepG2 cells were co-transfected with HBV DNA and a miR-204 expression vector. Reduction of intracellular HBV capsid particles were detected by native agarose gel electrophoresis and Western blot analysis. *middle panel*: The reduction in capsid particles was not due to the reduction of HBc protein, as assayed by denaturing SDS-PAGE and Western blot analysis. *lower panel*: tubulin served as a sample loading control. (**C**) Core particle-associated RNAs of HBV polymerase mutant Y63D, with or without miR-204 cotransfection, were analyzed by ribonuclease protection assay (RPA). Cotransfection with miR-204 reduced the levels of encapsidated HBV RNAs in HepG2. The protected HBV double-strand RNA fragment migrated as a 330 nt species on PAGE.

It was puzzling that miR-204 could reduce HBV DNA replication without any apparent reductions in HBV specific RNA and proteins (Fig. 2). We reasoned that a potential target for miR-204 could be at the step of RNA encapsidation and capsid assembly (Fig. 5A). To address this issue, we cotransfected HepG2 cells with HBV plasmid and miR-204 expression vector and examined the assembly efficiency and stability of intracellular core particles of HBV by native agarose gel (Fig. 5B *upper panel*). As a control, we also monitored the level of core protein monomer by denaturing SDS-PAGE (Fig. 5B, *lower panel*). While miR-204 significantly reduced the level of intracellular core particles, it had no effect on the total amount of core protein by SDS-PAGE. This result supports the notion that miR-204 could interfere with HBV capsid assembly. We also compared the efficiencies of pgRNA encapsidation with and without miR-204 cotransfection, by ribonuclease protection assay (RPA) using core particle-associated HBV RNA (Fig. 5C). Cotransfection with miR-204 reduced core particle-associated pgRNA, yet without any apparent effect on total cytoplasmic pgRNA. Our results suggest that miR-204 might target an unknown host factor(s) involved in capsid assembly or RNA encapsidation.

## Discussion

In this study, we reported two different mechanisms of two cellular miRNAs for their control over HBV replication and gene expression. The microRNA, miR-1236, was found to target a sequence localized within the 3′UTR of HBV specific RNAs, leading to reduced viral gene expression. The other microRNA, miR-204, inhibited HBV pregenomic RNA encapsidation and capsid assembly.

MiR-204 is generated from the sixth intron of the transient receptor potential melastatin 3 (TRPM3) gene^18^. We found miR-204 is expressed in multiple tissues in rats, with the highest level of expression in the eye and cerebellum by stem-loop real time PCR (Fig. S2). The expression level in the rat liver is low. It is possible that miR-204 might target an unknown host factor (s) involved in capsid assembly or RNA encapsidation. At present, approximately 400 validated targets of miR-204 have been documented in literature^19^. There is no easy way to speculate on which known or unknown miR-204 target might be involved in HBV RNA encapsidation and capsid assembly. Interestingly, this microRNA is well known for its important role in the eye retina development, adipogenesis, diabetes, and cancer^20^. In fact, miR-204 is being considered as a tumor suppressor. Since chronic infection with HBV can increase the risk of HCC development, it is possible that chronic suppression of miR-204 by HBV might contribute to the increased HCC incidence in chronic hepatitis B.

Previously, it has been demonstrated that treatment by interleukin-6 (IL-6) and epidermal growth factor stimulated the interaction between HBV enhancer 1 and STAT3 protein, which leads to the activation of HBV gene expression^21^. Therefore, we proposed here that HBV infection can activate the phosphorylation of STAT3, which in turn repressed the expression of miR-204, and thus stimulated HBV gene expression and replication. In this scenario, how STAT3 can suppress the level of miR-204 remains unclear. It is not mutually exclusive that the activated STAT3 can upregulate HBV transcription through its interaction with HBV enhancer 1, and at the same time, repress miR-204 expression. To further study on the regulation of miR-204 will provide more information about the control of HBV replication.

MiR-1236 is an intronic miRNA with no other family member (miRBase version 21). Interestingly, miR-1236 is found only in humans, pantroglodytes and pongopygmaeus, but not in rodents (GeneCards and miRBase version 21). We have no results on the tissue distribution of human miR-1236 at present. However, expression of miR-1236 can be found in various tissues including liver^22^. Previous studies showed that miR-1236 can repress VEGFR-3, RORγ, p21 promoter, and ZEB1 to inhibit inflammatory lymphangiogenesis, ulcerative colitis, cell proliferation, or cell migration/invasion, respectively^23^,^24^,^25^. In addition, miRNA-1236 contributes to HIV-1 restriction in monocytes^26^, and has a regulatory role in alpha-fetoprotein (AFP) expression and HCC development in the liver^22^. Recently, miR-1236 has also been shown to be induced by IL-1β and repressed by Twist1 to regulate the inflammatory lymphangiogenesis^23^ and hypoxia-induced EMT^27^. In our study, we discovered a novel role of miR-1236 in inhibiting HBV replication by direct targeting at HBV specific RNA. The successful bioinformatics approach to identifying miR-1236 allows us to anticipate that miR-3960, miR-3166, miR-4763, miR-665 and miR-663, are potential candidates for anti-HBV miRNA (Tables 1 and 2).

To date, at least three miRNA candidates with therapeutic potential have been selected into clinical trials^28^,^29^. One well known example is Miravirsen, a locked nucleic acid–modified (LNA) DNA phosphorothioate antisense oligonucleotide. It can sequester mature miR-122, which is essential to HCV replication. The treatment of Miravirsen in chronic HCV infected patients showed prolonged and dose-dependent repression of HCV RNA level without any apparent adverse effects or viral resistance^29^. The success example of Miravirsen on HCV would certainly encourage a similar miRNA approach for HBV or other viral diseases in the future.

In summary, instead of adopting an evasion strategy like target site mutations, HBV could take an alternative strategy by reducing the expression of hostile cellular microRNAs. Since the effects of miR-1236 and miR-204 on HBV appeared to be independent of HBV genotypes, their therapeutic potential may be broad spectrum, and worth further investigation.

## Material and Methods

### Ethics Statement

All animal experiments were conducted under protocols approved by Academia Sinica Institutional Animal Care & Utilization Committee (ASIACUC). Research was conducted in compliance with the principles stated in the Guide for the Care and Use of Laboratory Animals, National Research Council, 1996.

### Animals

The generation of HBV transgenic mice on an ICR background has been reported previously^10^. The transgene is a 1.3-fold HBV genome (genotype D, serotype *ayw*). The Tg[HBV1.3] mouse line was used in this study. All animals were housed in a specific-pathogen-free environment in the animal facility of the Institute of Biomedical Sciences, Academia Sinica, Taiwan.

### Cell Culture

Human hepatoma HuH-7^30^, HepG2^31^ and rat hepatoma cell line Q7 cells^8^ were maintained as described previously^11^,^12^,^13^,^14^,^32^. Stable Qs2, Qs4, Qs5, and Qs21 cell lines can produce infectious HBV and were established by transfecting Q7 (Morris hepatoma 7777) cells with a genomic dimer of HBV DNA (*ayw*)^8^,^9^. Stable UP7-4 and UP7-7cell lines were established by transfecting human HepG2 cells with a genomic dimer of HBV DNA (*ayw*)^33^. While the phenotypic effects of microRNAs on viral replication are in general stronger in HepG2 than HuH-7 cells, the latter are less sticky, easier to trypsinize and grow in culture. Therefore, we used both cell lines with no preference.

### Construction of miRNA Plasmids

The methods of engineering the expression vectors of miR-204 and miR-1236 were as described previously^5^,^33^. Briefly, the sequences of human miR-204 and miR-1236 were retrieved from Ensembl database and miRBase (Version 16). Human genomic DNA extracted from HepG2 cells was used as a template for PCR amplification of precursor sequences of miR-204 and miR-1236. The PCR primers for cloning: pre-hsa-miR-204-F 5′-GAGGGCCTCCTGATCATTTACC-3′; pre-hsa-miR-204-R 5′-CCAGAGCTGCTTGGATGAA-3′; pre-hsa-miR-1236-F 5′-GCAAAGTAGCAGCAGCACAA-3′, pre-hsa-miR-1236-R 5′-GTTGCTGTGGCTGTGTCCAT-3′. All plasmids were confirmed by sequencing.

### Quantitative Real-time PCR

Briefly, 2 μg of total RNA was reverse transcribed into cDNA at 37 °C for 2 hours using random primers and High Capacity cDNA Reverse Transcription kit (Applied Biosystem, Grand Island, NY). The cDNA product was analyzed by 1.5% agarose gel electrophoresis and visualized by staining with HealthView (Genomics BioSci & Tech, Taiwan). The cDNA product was then diluted 100 times for real-time PCR analysis using Power SYBR Green PCR master mix (Applied Biosystem, Grand Island, NY), and the default condition in a 20 μl reaction volume by Applied Biosystems 7500 Real-Time PCR System. Data were analyzed by relative quantification methods (∆∆Ct methods) using 7500 software V2.0.1.

### Stem-loop qPCR for miRNA

Taqman RT and stem-loop real-time assay were from Applied Biosystems: miR-31 (assayID: 002279), miR-130a (assayID: 000454), miR-204-5p (assayID: 000508) and miR-1236 (assayID: 002761). Briefly, 100 ng RNAs were reverse transcribed by specific stem-loop primer and further analyzed by Taqman real-time PCR assay using default setting. U6 snRNA (assayID: 001973) was used as an internal loading control. Data were analyzed by Applied Biosystems 7500 software V2.0.1. MiR-204 hairpin precursor can generate both miR-204-5p (miR-204) and its complementary miR-204-3p (miR-204*) from the other arm. We focused on miR-204-5p here because the LNA experiment (Fig. 2D–F) confirmed the anti-HBV potential from miR-204-5p. In this report, we used the old name miR-204 and the new name miR-204-5p interchangeably.

### Treatment with STAT3 inhibitor

STAT inhibitor (S31-201) was purchased from Selleckchem. HepG2 cells were seeded in 6-well tissue culture plates at 5 × 10^5^ cells/well and cultured at 37 °C for 24 hrs. S31-201 was prepared in 0.1% DMSO and added to culture medium at indicated concentrations. Culture medium was changed every two days before harvest.

### Sources of antibodies

Vendors for antibodies are shown in parentheses: anti-HBc (Dako, Real Carpinteria, CA), anti-STAT3 (GeneTex, Taiwan), anti-GAPDH, anti-tubulin and anti-phospho-Stat3 (tyr705) (Cell Signaling Technology Inc, Danvers, MA). Secondary antibodies include mouse anti-rabbit-HRP, goat anti-mouse-HRP (GeneTex, Taiwan).

### Southern and Northern blots

HBV core particle-associated DNA and total cellular cytoplasmic mRNAs were analyzed by Southern and Northern blots as described previously^34^. Each lane on the Southern blot gel was loaded with the total amount of core particle-associated viral DNA extracted from each transfected dish, which was seeded with equal cell density one night before transfection.

### Native agarose gel electrophoresis and Western blot of HBV core particles

HBV core particles and total HBV core protein were analyzed by native agarose gel electrophoresis and Western blot as described previously^34^.

### The 3′UTR luciferase reporter assay

The indicated 3′UTRs of HBV RNA genome were amplified from HBV *ayw* dimer genome plasmid using their respective forward and reverse primers (Forward: 5′-AGCAGGTCTGGAGCAAACAT-3′; Reverse: 5′-CACCCACCCAGGTAGCTAGA-3′), and cloned into a psiCHECK-2 luciferase vector (Promega, Madison, WI). Target site mutants containing altered sequences at HBV 3′UTR and a miR-1236 mutant containing altered seed sequences, were engineered by using paired mutant primers (HBV mutant 3′UTR Forward: 5′-GGAGGAGTTGGGAGAGGAAATTAGGTTAAAGG-3′; Reverse: 5′-CCTTTAACCTAATTTCCTCTCCCAACTCCTCC-3′, miR-1236 mutant Forward: 5′-GCCAACATAATGCTTCTTCTCCTTGTCTCTCC-3′; Reverse: 5′-GGAGAGACAAGGAGAAGAAGCATTATGTTGGC-3′) and Site-directed Mutagenesis Kit (Stratagene, Santa Clara, CA). Mutants were identified by DNA sequencing.

### Bioinformatic analysis

Computer-based programs including Targetscan (http://www.targetscan.org/), Pictar (http://pictar.mdc-berlin.de/), Microinspector (http://bioinfo.uni-plovdiv.bg/microinspector/), RNAhybrid (http://www. bibiserv.techfak.uni-bielefeld.de/) and DIANA (http://diana.cslab.ece.ntua.gr) were used to predict potential targets for miR-1236 and miR-204 on HBV genome. The minimal free energy of binding less than −20 kcal/mol was used as the cut-off value.

### RNase protection analysis (RPA)

RPA was performed using the vendor’s protocol (RPA III, Ambion). A 392 nt antisense riboprobe was radiolabelled by *in vitro* transcription using a *NotI* linearized DNA fragment from a pGEM-T vector containing HBV sequences nt 2150–1820. RNase would preferentially digest single-strand RNA, but has no significant effect on the hybridized double-strand RNA. The protected pgRNA fragment was 330 nt in length. HBV polymerase mutant Y63D is defective in DNA synthesis, yet competent in pgRNA encapsidation^35^,^36^. This mutant accumulates a higher level of encapsidated pgRNA in the core particles due to its arrested priming of cDNA synthesis.

### Statistics

Statistical significance was determined using the Student’s *t* test. In all figures, values were expressed as mean ± standard deviation (SD) and statistical significance (*p* < 0.05) was indicated by an asterisk. The data represent results from at least three independent experiments.

## Additional Information

**How to cite this article**: Huang, J.-Y. *et al*. MicroRNA miR-204 and miR-1236 inhibit hepatitis B virus replication via two different mechanisms. *Sci. Rep.* **6**, 34740; doi: 10.1038/srep34740 (2016).

## Supplementary Material

Supplementary Information

## Figures and Tables

**Table 1:** Computational prediction of cellular microRNAs with a strong potential of binding to HBV 3′UTR RNA#.

miRNA	Free energy (kcal/mol)	Target position@
**hsa-miR-1236**	**−30.8**	**nt 1724**
hsa-miR-3960	−30.3	nt 1579
hsa-miR-3166	−29.9	nt 1774
hsa-miR-663	−29.5	nt 1525
hsa-miR-638	−27.6	nt 1521
hsa-miR-3197	−27.3	nt 1535
hsa-miR-1207-5p	−27.3	nt 1543
hsa-miR-939	−27.3	nt 1522
hsa-miR-4763-3p	−27.2	nt 1542
hsa-miR-663	−27.2	nt 1562
hsa-miR-4783-3p	−26.9	nt 1990
hsa-miR-3183	−26.8	nt 1722
hsa-miR-2861	−26.41	nt 1621
hsa-miR-3943	−26.3	nt 1869
hsa-miR-939	−25.9	nt 1521
hsa-miR-663b	−25.9	nt 1590
hsa-miR-4689	−25.8	nt 1537
hsa-miR-574-5p	−25.4	nt 2036
hsa-miR-665	−25.1	nt 2009

^#^The entire miRBase version 21.0 were screened with HBV (*ayw*) mRNA sequences (nt 1521 – 2122) using the Microinspector Program.

^@^The positions refer to the first nucleotide of microRNA binding sites on HBV ayw genome.

**Table 2:** Computational prediction of cellular microRNAs with a strong potential of binding to whole HBV RNA pregenome#.

miRNA	Free energy (kcal/mol)	Target position
hsa-miR-665	−35.6	nt641
hsa-miR-3960	−34.4	nt1478
hsa-miR-4763-3p	−33.6	nt1147
hsa-miR-4707-5p	−33.1	nt179
hsa-miR-874	−32.7	nt3035
hsa-miR-1538	−31.4	nt2979
hsa-miR-3940-3p	−31.4	nt2979
**hsa-miR-1236**	−**30.8**	**nt1724**
hsa-miR-1207-5p	−30.7	nt1233
hsa-miR-663b	−30.6	nt1198
hsa-miR-4783-3p	−30.6	nt1181
hsa-miR-4685-5p	−30.5	nt593
hsa-miR-3960	−30.3	nt1578
hsa-miR-1291	−30.1	nt1196
hsa-miR-3065-3p	−30	nt346

^@^The positions refer to the first nucleotide of microRNA binding sites on HBV *ayw* genome.

^#^The entire miRbase version 21.0 were screened with HBV (*ayw*) whole genome sequences using the Microinspector Program.
