# Supplementary material for: MicroRNA miR-204 and miR-1236 inhibit hepatitis B virus replication via two different mechanisms
Source: Sci Rep. 2016 Oct 13;6:34740. doi: 10.1038/srep34740 (PMC5062086; doi:10.1038/srep34740)
Supplement: Supplementary Information [file srep34740-s1.pdf]

Supplementary Information

MicroRNA miR-204 and miR-1236 inhibit hepatitis B virus replication via two different mechanisms

Running Title: molecular mechanisms of anti-HBV microRNAs

Authors: Jyun-Yuan Huang[1], Hung-Lin Chen[1], and Chiaho Shih[1*]

[1]Institute of Biomedical Sciences, Academia Sinica, Taipei, Taiwan

*To whom correspondence should be addressed

Mailing address: Institute of Biomedical Sciences, Academia Sinica, Taipei, Taiwan

Tel: 886-2-2652-3996; Fax: 8862-2652-3597; E-mail: cshih@ibms.sinica.edu.tw

Fig. S1

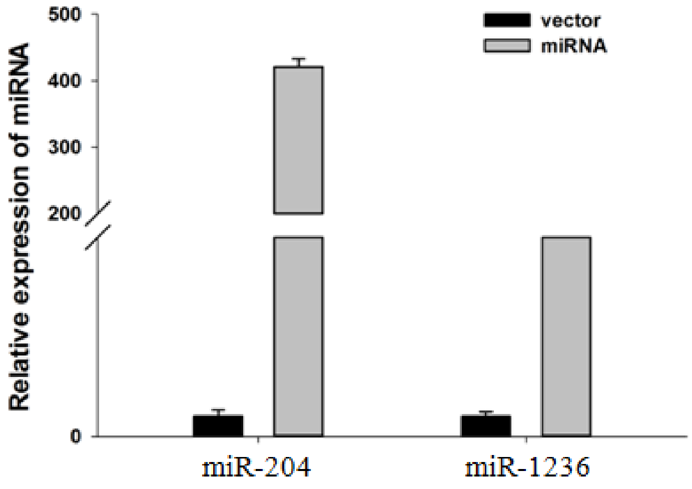

Fig. S1 The expressions of miR-204 and miR-1236 in transient cotransfection were detected by stem-loop PCR analysis. In the cotransfection experiment described in Fig. 2, expression vectors of miR-204 or miR-1236 were contransfected with HBV (*ayw*) genomic dimer DNA into HepG2 cells, respectively. Expression of the vector control = 1.

Fig. S2

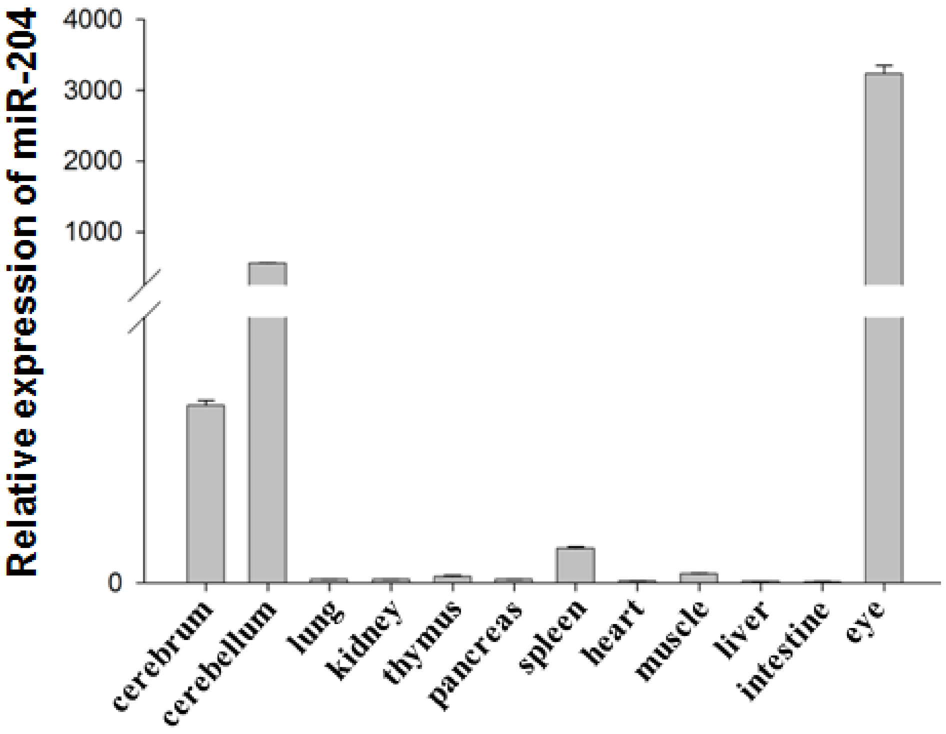

Fig. S2 MiR-204 is expressed in multiple rat tissues, with the highest level of expression in eye and cerebellum. The expression levels in the rat liver, kidney, lung, heart, intestine and pancreas are low. The expression level of muscle is used here as a reference.
